# *N*,*N*-Dimethyl­acetamide–4-iodo­benzene­sulfonic acid–water (1/1/1)

**DOI:** 10.1107/S160053680803701X

**Published:** 2008-11-20

**Authors:** Rui Liu, Yu-Feng Li, Wei Luo, Jin Chang, Hong-Jun Zhu

**Affiliations:** a Department of Applied Chemistry, College of Science, Nanjing University of Technology, Nanjing 210009, People’s Republic of China

## Abstract

In the title compound, C_6_H_5_IO_3_S·C_4_H_9_NO·H_2_O, *N*,*N*-dimethylacetamide and 4-iodobenzenesulfonic acidmolecules are linked by an intramolecular C—H⋯O hydrogen bond. In the crystal structure, inter­molecular O—H⋯O, O—H⋯I and C—H⋯O hydrogen bonds link the mol­ecules.

## Related literature

For a related structure, see: Wu *et al.* (2000 ▶). For bond-length data, see: Allen *et al.* (1987 ▶).
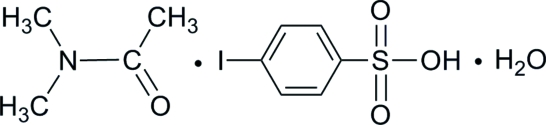


## Experimental

### 

#### Crystal data

C_6_H_5_IO_3_S·C_4_H_9_NO·H_2_O
*M* *_r_* = 389.21
Orthorhombic, 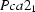

*a* = 14.173 (3) Å
*b* = 7.7480 (15) Å
*c* = 13.272 (3) Å
*V* = 1457.4 (5) Å^3^
*Z* = 4
Mo *K*α radiation
μ = 2.35 mm^−1^
*T* = 294 (2) K
0.30 × 0.20 × 0.10 mm

#### Data collection

Enraf–Nonius CAD-4 diffractometer
Absorption correction: ψ scan (North *et al.*, 1968 ▶) *T*_min_ = 0.539, *T*_max_ = 0.799
1490 measured reflections
1490 independent reflections
1096 reflections with *I* > 2σ(*I*)
3 standard reflections frequency: 120 min intensity decay: none

#### Refinement

*R*[*F* ^2^ > 2σ(*F* ^2^)] = 0.063
*wR*(*F* ^2^) = 0.162
*S* = 1.07
1490 reflections
172 parameters
4 restraints
H atoms treated by a mixture of independent and constrained refinement
Δρ_max_ = 1.53 e Å^−3^
Δρ_min_ = −2.42 e Å^−3^
Absolute structure: Flack (1983 ▶), 7 Friedel pairs
Flack parameter: 0.13 (10)

### 

Data collection: *CAD-4 Software* (Enraf–Nonius, 1985 ▶); cell refinement: *CAD-4 Software*; data reduction: *XCAD4* (Harms & Wocadlo, 1995 ▶); program(s) used to solve structure: *SHELXS97* (Sheldrick, 2008 ▶); program(s) used to refine structure: *SHELXL97* (Sheldrick, 2008 ▶); molecular graphics: *PLATON* (Spek, 2003 ▶); software used to prepare material for publication: *SHELXTL* (Sheldrick, 2008 ▶).

## Supplementary Material

Crystal structure: contains datablocks I, global. DOI: 10.1107/S160053680803701X/hk2535sup1.cif

Structure factors: contains datablocks I. DOI: 10.1107/S160053680803701X/hk2535Isup2.hkl

Additional supplementary materials:  crystallographic information; 3D view; checkCIF report

## Figures and Tables

**Table 1:** Hydrogen-bond geometry (Å, °)

*D*—H⋯*A*	*D*—H	H⋯*A*	*D*⋯*A*	*D*—H⋯*A*
O1*W*—H1*WA*⋯O2^i^	0.87 (13)	1.97 (15)	2.765 (16)	151 (14)
O1*W*—H1*WB*⋯O3^ii^	0.94 (10)	1.85 (15)	2.657 (16)	143 (17)
O2—H2*A*⋯I1^iii^	0.85	2.57	3.208 (16)	133
C1—H1*B*⋯O3^iv^	0.93	2.46	3.378 (15)	168
C5—H5*A*⋯O1^v^	0.93	2.55	3.192 (17)	126
C9—H9*A*⋯O3	0.96	2.56	3.48 (2)	161

Symmetry codes: (i) 
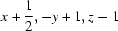
; (ii) 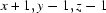; (iii) 
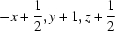
; (iv) 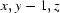; (v) 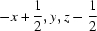.

**Fig. 1.**
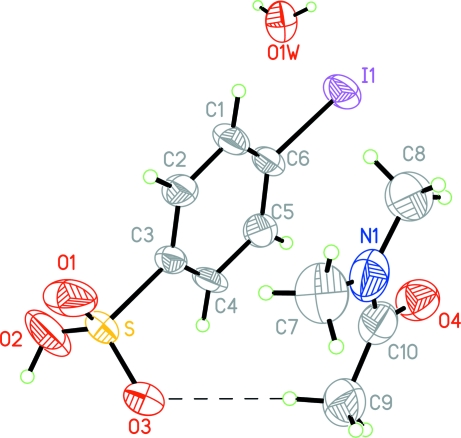
The molecular structure of the title molecule, with the atom-numbering scheme. Displacement ellipsoids are drawn at the 50% probability level. Hydrogen bonds are shown as dashed lines.

**Fig. 2.**
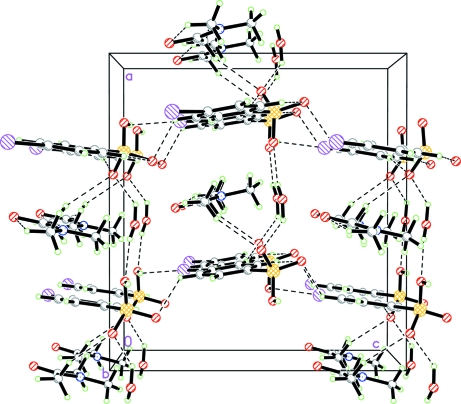
A packing diagram of the title compound. Hydrogen bonds are shown as dashed lines.

**Fig. 3.**
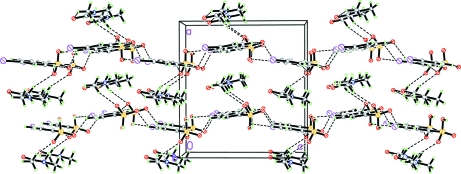
A packing diagram of the title compound, showing the formation of the supramolecular comb-like structure. For the sake of clarity, water molecules have been omitted.

## supplementary crystallographic information

### Comment

The crystal structure of the title compound with a comb-like structure illustrate the three different components linked by weak interactions based on hydrogen bonds. Furthermore, the hydrolysis mechanism of the innersalt, which was formed from 4-iodobenzenesulfonyl chloride and N,N-dimethylacetamide, was understood (Wu *et al.*,  2000). Meanwhile, the complicated hydrolysate was finally confirmed. We report herein its crystal structure.

The asymmetric unit of the title compound contains N,N-dimethylacetamide, 4-iodobenzenesulfonic acid and water molecules (Fig. 1), in which the bond lengths (Allen *et al.*,  1987) and angles are within normal ranges. Ring A (C1-C6) is, of course, planar. The intramolecular C-H···O hydrogen bonds (Table 1) result in the formation of two nonplanar five-membered rings B (S/O1/C2/C3/H2B) and C (O4/N1/C8/C10/H8A), having envelope conformations with O1 and H8A atoms displaced by 0.193 (3) and 0.194 (3) Å, respectively, from the planes of the other ring atoms.

In the crystal structure, intermolecular O-H···O, O-H···I and C-H···O hydrogen bonds link the molecules (Fig. 2),  in which they may be effective in the stabilization of the structure. As can be seen from the packing diagram (Fig. 3), the molecules are stacked along the b axis. The comb-like structure depends on C-H···O hydrogen bonds. The 4-iodobenzenesulfonic acid molecules constitute the main chain and the N,N-dimethylacetamide molecules intermesh to each other as the branches.

### Experimental

Addition of N,N-dimethylacetamide (1.8 ml, 0.02 mol) into 4-iodobenzenesulfonyl chloride (6.1 g, 0.02 mol) gave milk-white solution of innersalt (Wu *et al.*,  2000). The innersalt was dissolved in acetone (20 ml) and placed in moist chamber to crystallize. The crystals were obtained by evaporating solvent slowly at room temperature for about 40 d.

### Refinement

Water H atoms were located in difference syntheses and refined as [O-H = 0.88 (9) Å and 0.94 (9) Å; U_iso_(H) = 0.093 Å^2^]. The remaining H atoms were positioned geometrically, with O-H = 0.85 Å (for OH) and C-H = 0.93 and 0.96 Å for aromatic and methyl H, respectively, and constrained to ride on their parent atoms with U_iso_(H) = xU_eq_(C,O), where x = 1.5 for methyl H and x = 1.2 for all other H atoms.

### Crystal data

**Table:**

C_6_H_5_IO_3_S·C_4_H_9_NO·H_2_O	*D*_x_ = 1.774 Mg m^−^^3^
*M**_r_* = 389.21	Melting point: 363 K
Orthorhombic, *P**c**a*2_1_	Mo *K*α radiation λ = 0.71073 Å
Hall symbol: P 2c -2ac	Cell parameters from 25 reflections
*a* = 14.173 (3) Å	θ = 10–13º
*b* = 7.7480 (15) Å	µ = 2.35 mm^−^^1^
*c* = 13.272 (3) Å	*T* = 294 (2) K
*V* = 1457.4 (5) Å^3^	Block, colorless
*Z* = 4	0.30 × 0.20 × 0.10 mm
*F*_000_ = 768	

### Data collection

**Table:**

Enraf–Nonius CAD-4 diffractometer	*R*_int_ = 0.0000
Radiation source: fine-focus sealed tube	θ_max_ = 25.9º
Monochromator: graphite	θ_min_ = 2.6º
*T* = 294(2) K	*h* = 0→17
ω/2θ scans	*k* = 0→9
Absorption correction: ψ scan(North *et al.*, 1968)	*l* = 0→16
*T*_min_ = 0.539, *T*_max_ = 0.799	3 standard reflections
1490 measured reflections	every 120 min
1490 independent reflections	intensity decay: none
1096 reflections with *I* > 2σ(*I*)	

### Refinement

**Table:**

Refinement on *F*^2^	Hydrogen site location: inferred from neighbouring sites
Least-squares matrix: full	H atoms treated by a mixture of independent and constrained refinement
*R*[*F*^2^ > 2σ(*F*^2^)] = 0.063	*w* = 1/[σ^2^(*F*_o_^2^) + (0.1045*P*)^2^] where *P* = (*F*_o_^2^ + 2*F*_c_^2^)/3
*wR*(*F*^2^) = 0.162	(Δ/σ)_max_ < 0.001
*S* = 1.07	Δρ_max_ = 1.53 e Å^−^^3^
1490 reflections	Δρ_min_ = −2.42 e Å^−^^3^
172 parameters	Extinction correction: none
4 restraints	Absolute structure: Flack (1983), 7 Friedel pairs
Primary atom site location: structure-invariant direct methods	Flack parameter: 0.13 (10)
Secondary atom site location: difference Fourier map	

### Special details

**Table:**

Geometry. All e.s.d.'s (except the e.s.d. in the dihedral angle between two l.s. planes) are estimated using the full covariance matrix. The cell e.s.d.'s are taken into account individually in the estimation of e.s.d.'s in distances, angles and torsion angles; correlations between e.s.d.'s in cell parameters are only used when they are defined by crystal symmetry. An approximate (isotropic) treatment of cell e.s.d.'s is used for estimating e.s.d.'s involving l.s. planes.
Refinement. Refinement of *F*^2^ against ALL reflections. The weighted *R*-factor *wR* and goodness of fit *S* are based on *F*^2^, conventional *R*-factors *R* are based on *F*, with *F* set to zero for negative *F*^2^. The threshold expression of *F*^2^ > σ(*F*^2^) is used only for calculating *R*-factors(gt) *etc*. and is not relevant to the choice of reflections for refinement. *R*-factors based on *F*^2^ are statistically about twice as large as those based on *F*, and *R*- factors based on ALL data will be even larger.

### Fractional atomic coordinates and isotropic or equivalent isotropic displacement parameters (Å^2^)

**Table:**

	*x*	*y*	*z*	*U*_iso_*/*U*_eq_	
I1	0.20688 (5)	0.25040 (11)	0.7328 (2)	0.0505 (3)	
S	0.1876 (2)	0.9056 (4)	1.0531 (3)	0.0438 (8)	
O1W	0.9586 (8)	0.1582 (19)	0.0789 (10)	0.077 (4)	
H1WA	0.914 (10)	0.084 (19)	0.065 (17)	0.093*	
H1WB	1.023 (7)	0.13 (2)	0.082 (15)	0.093*	
O1	0.1588 (10)	0.8396 (14)	1.1505 (8)	0.076 (4)	
O2	0.2802 (7)	0.9782 (15)	1.0512 (14)	0.094 (5)	
H2A	0.2768	1.0848	1.0659	0.113*	
O3	0.1189 (8)	1.0230 (11)	1.0115 (8)	0.053 (2)	
O4	−0.0108 (7)	0.6431 (14)	0.7131 (8)	0.060 (3)	
N1	−0.0502 (9)	0.597 (2)	0.8736 (12)	0.070 (4)	
C1	0.1840 (9)	0.4229 (16)	0.9376 (10)	0.041 (3)	
H1B	0.1756	0.3110	0.9613	0.050*	
C2	0.1806 (9)	0.5632 (16)	1.0022 (10)	0.043 (3)	
H2B	0.1709	0.5450	1.0706	0.051*	
C3	0.1912 (8)	0.7263 (14)	0.9674 (11)	0.034 (3)	
C4	0.2059 (8)	0.7609 (14)	0.8682 (12)	0.038 (3)	
H4A	0.2132	0.8741	0.8461	0.046*	
C5	0.2100 (8)	0.6222 (17)	0.7992 (10)	0.044 (3)	
H5A	0.2188	0.6412	0.7306	0.053*	
C6	0.2002 (8)	0.4547 (15)	0.8381 (10)	0.038 (3)	
C7	−0.0714 (13)	0.648 (3)	0.9756 (12)	0.078 (5)	
H7A	−0.1176	0.7386	0.9748	0.117*	
H7B	−0.0149	0.6890	1.0076	0.117*	
H7C	−0.0956	0.5509	1.0121	0.117*	
C8	−0.0515 (12)	0.407 (2)	0.8500 (15)	0.073 (5)	
H8A	−0.0490	0.3903	0.7784	0.110*	
H8B	−0.1084	0.3562	0.8760	0.110*	
H8C	0.0020	0.3519	0.8808	0.110*	
C9	−0.0327 (13)	0.897 (2)	0.8184 (15)	0.072 (5)	
H9A	0.0203	0.9300	0.8590	0.107*	
H9B	−0.0901	0.9276	0.8522	0.107*	
H9C	−0.0296	0.9548	0.7546	0.107*	
C10	−0.0305 (12)	0.705 (2)	0.8017 (15)	0.062 (4)	

### Atomic displacement parameters (Å^2^)

**Table:**

	*U*^11^	*U*^22^	*U*^33^	*U*^12^	*U*^13^	*U*^23^
I1	0.0651 (5)	0.0364 (4)	0.0499 (5)	0.0024 (4)	−0.0001 (8)	−0.0136 (4)
S	0.0544 (17)	0.0296 (14)	0.0473 (17)	0.0041 (14)	−0.0096 (18)	−0.0142 (15)
O1W	0.054 (6)	0.103 (10)	0.075 (9)	0.002 (7)	0.005 (6)	−0.025 (8)
O1	0.141 (11)	0.047 (6)	0.039 (6)	0.028 (7)	0.002 (6)	−0.003 (5)
O2	0.076 (8)	0.059 (7)	0.147 (13)	0.004 (6)	−0.020 (9)	−0.062 (9)
O3	0.062 (6)	0.035 (5)	0.060 (6)	0.008 (4)	−0.006 (5)	−0.011 (4)
O4	0.065 (6)	0.060 (6)	0.055 (7)	0.006 (5)	0.000 (5)	0.001 (6)
N1	0.052 (8)	0.083 (10)	0.074 (10)	−0.008 (8)	−0.016 (7)	0.003 (9)
C1	0.057 (7)	0.024 (6)	0.043 (7)	−0.009 (6)	0.001 (6)	0.000 (5)
C2	0.064 (8)	0.034 (7)	0.030 (6)	0.003 (6)	−0.006 (6)	0.003 (6)
C3	0.031 (6)	0.025 (6)	0.045 (7)	0.004 (5)	−0.002 (5)	−0.006 (5)
C4	0.049 (7)	0.017 (5)	0.048 (8)	−0.001 (5)	0.000 (6)	−0.003 (5)
C5	0.051 (8)	0.042 (7)	0.039 (7)	0.006 (6)	−0.004 (6)	−0.003 (6)
C6	0.049 (7)	0.024 (6)	0.041 (7)	−0.002 (5)	−0.002 (6)	−0.006 (6)
C7	0.082 (12)	0.101 (15)	0.051 (10)	−0.005 (11)	0.018 (9)	−0.004 (10)
C8	0.061 (10)	0.070 (11)	0.089 (13)	−0.011 (9)	0.000 (9)	0.008 (11)
C9	0.078 (12)	0.079 (13)	0.059 (10)	0.013 (9)	−0.012 (9)	−0.017 (10)
C10	0.060 (10)	0.060 (10)	0.066 (11)	−0.002 (8)	−0.018 (9)	0.009 (9)

### Geometric parameters (Å, °)

**Table:**

I1—C6	2.113 (12)	C2—H2B	0.9300
S—O2	1.429 (11)	C3—C4	1.36 (2)
S—O3	1.441 (10)	C4—C5	1.413 (18)
S—O1	1.449 (12)	C4—H4A	0.9300
S—C3	1.796 (12)	C5—C6	1.404 (18)
O1W—H1WA	0.88 (9)	C5—H5A	0.9300
O1W—H1WB	0.94 (9)	C7—H7A	0.9600
O2—H2A	0.8500	C7—H7B	0.9600
O4—C10	1.30 (2)	C7—H7C	0.9600
N1—C10	1.30 (2)	C8—H8A	0.9600
N1—C7	1.44 (2)	C8—H8B	0.9600
N1—C8	1.51 (2)	C8—H8C	0.9600
C1—C6	1.363 (19)	C9—C10	1.50 (2)
C1—C2	1.386 (17)	C9—H9A	0.9600
C1—H1B	0.9300	C9—H9B	0.9600
C2—C3	1.354 (17)	C9—H9C	0.9600
			
O2—S—O3	111.4 (8)	C4—C5—H5A	121.3
O2—S—O1	114.4 (9)	C1—C6—C5	122.7 (12)
O3—S—O1	112.0 (7)	C1—C6—I1	120.8 (9)
O2—S—C3	105.5 (6)	C5—C6—I1	116.4 (9)
O3—S—C3	105.3 (6)	N1—C7—H7A	109.5
O1—S—C3	107.5 (7)	N1—C7—H7B	109.5
H1WA—O1W—H1WB	125 (10)	H7A—C7—H7B	109.5
S—O2—H2A	109.0	N1—C7—H7C	109.5
C10—N1—C7	123.8 (18)	H7A—C7—H7C	109.5
C10—N1—C8	118.8 (16)	H7B—C7—H7C	109.5
C7—N1—C8	117.5 (17)	N1—C8—H8A	109.5
C6—C1—C2	117.6 (12)	N1—C8—H8B	109.5
C6—C1—H1B	121.2	H8A—C8—H8B	109.5
C2—C1—H1B	121.2	N1—C8—H8C	109.5
C3—C2—C1	121.2 (13)	H8A—C8—H8C	109.5
C3—C2—H2B	119.4	H8B—C8—H8C	109.5
C1—C2—H2B	119.4	C10—C9—H9A	109.5
C2—C3—C4	122.1 (12)	C10—C9—H9B	109.5
C2—C3—S	120.2 (11)	H9A—C9—H9B	109.5
C4—C3—S	117.7 (9)	C10—C9—H9C	109.5
C3—C4—C5	118.9 (11)	H9A—C9—H9C	109.5
C3—C4—H4A	120.5	H9B—C9—H9C	109.5
C5—C4—H4A	120.5	O4—C10—N1	118.1 (16)
C6—C5—C4	117.5 (13)	O4—C10—C9	120.3 (16)
C6—C5—H5A	121.3	N1—C10—C9	121.7 (19)
			
C6—C1—C2—C3	1(2)	S—C3—C4—C5	179.4 (8)
C1—C2—C3—C4	0(2)	C3—C4—C5—C6	−1.2 (17)
C1—C2—C3—S	−179.4 (10)	C2—C1—C6—C5	−2(2)
O2—S—C3—C2	114.1 (12)	C2—C1—C6—I1	180.0 (9)
O3—S—C3—C2	−127.9 (11)	C4—C5—C6—C1	2.3 (18)
O1—S—C3—C2	−8.3 (13)	C4—C5—C6—I1	−179.9 (8)
O2—S—C3—C4	−65.0 (13)	C7—N1—C10—O4	178.7 (14)
O3—S—C3—C4	52.9 (11)	C8—N1—C10—O4	−1(2)
O1—S—C3—C4	172.5 (10)	C7—N1—C10—C9	−3(3)
C2—C3—C4—C5	0.3 (19)	C8—N1—C10—C9	176.9 (14)

### Hydrogen-bond geometry (Å, °)

**Table:**

*D*—H···*A*	*D*—H	H···*A*	*D*···*A*	*D*—H···*A*
O1W—H1WA···O2^i^	0.87 (13)	1.97 (15)	2.765 (16)	151 (14)
O1W—H1WB···O3^ii^	0.94 (10)	1.85 (15)	2.657 (16)	143 (17)
O2—H2A···I1^iii^	0.85	2.57	3.208 (16)	133
C1—H1B···O3^iv^	0.93	2.46	3.378 (15)	168
C2—H2B···O1	0.93	2.52	2.925 (17)	106
C5—H5A···O1^v^	0.93	2.55	3.192 (17)	126
C8—H8A···O4	0.96	2.21	2.64 (2)	106
C9—H9A···O3	0.96	2.56	3.48 (2)	161

Symmetry codes: (i) *x*+1/2, −*y*+1, *z*−1; (ii) *x*+1, *y*−1, *z*−1; (iii) −*x*+1/2, *y*+1, *z*+1/2; (iv) *x*, *y*−1, *z*; (v) −*x*+1/2, *y*, *z*−1/2.
